# Effects of Type of Concentrate and Timing of Supplementation on Feed Intake, Nitrogen Use, and Performance in Lactating Dairy Cows Grazing an Alfalfa-Ryegrass Sward

**DOI:** 10.3390/ani12101235

**Published:** 2022-05-11

**Authors:** Uta Dickhoefer, Pedro Alan Sainz-Sanchez, Gustavo Rojas, Joaquín Miguel Castro-Montoya, Carlos Gomez

**Affiliations:** 1 Animal Nutrition and Rangeland Management in the Tropics and Subtropics, University of Hohenheim, 70599 Stuttgart, Germany; alan.sainz@uni-hohenheim.de (P.A.S.-S.); grojas.or@gmail.com (G.R.); joaquin.montoya@ues.edu.sv (J.M.C.-M.); 2 Institute of Animal Nutrition and Physiology, Kiel University, 24118 Kiel, Germany; 3 Departamento Académico de Nutrición de la Facultad de Zootecnia, Universidad Nacional Agraria La Molina, Ap. 456, Lima 12, Peru; cagomez@lamolina.edu.pe

**Keywords:** ruminant, legume pasture, feeding behavior, rumen microbial protein synthesis, nitrogen balance

## Abstract

**Simple Summary:**

Supplementing non-structural carbohydrates can enhance feed intake, performance, and nitrogen use in dairy cows grazing protein-rich swards. The present study thus analyzed the effects of feeding lactating cows two types of cereal grains, when their majority was either offered before or after grazing an alfalfa-ryegrass sward. Results showed that supplementing corn meal as a slowly degradable starch source after grazing and oat meal as a rapidly degradable starch source before grazing may improve milk yield and nitrogen use in grazing dairy cows. Hence, matching the choice of concentrate feed and the timing of its supplementation may aid to reduce nitrogen emissions from pasture-based dairy cattle systems while making use of the local, human-inedible forage resources from grasslands.

**Abstract:**

The aim was to analyze the effects of two cereal grains differing in nutritional composition and starch degradation characteristics and the timing of their supplementation on feed intake, rumen microbial protein synthesis (MPS), performance, and nitrogen use of lactating dairy cows grazing an alfalfa-ryegrass sward. Four dietary treatments were tested in 24 lactating Brown Swiss cows in an incomplete 4 × 3 Latin square design. Cows were supplemented with 3.5 kg/d (as-fed basis) of a corn-based or an oat-based concentrate mixture (CM), of which either the majority (2.5 vs. 1.0 kg/d) was offered before or after grazing. Feed intake was similar across diets, but the interaction between type of CM and timing of supplementation affected eating time (*p* = 0.010), milk protein (*p* = 0.013) and energy-corrected milk yields (*p* = 0.025), efficiency of rumen MPS (*p* = 0.094), and nitrogen use efficiency (*p* = 0.081). Most of these variables were greater when the majority of the corn-based CM was offered after grazing and the oat-based CM before grazing. Supplementing slowly degradable starch sources after and rapidly degradable starch sources before grazing may improve the efficiency of rumen MPS, milk performance, and nitrogen use efficiency in dairy cows grazing alfalfa-ryegrass swards.

## 1. Introduction

Alfalfa is a high-quality forage legume commonly used in dairy cattle feeding as a source of protein. However, high proportions of crude protein (CP) that is rapidly degraded in the rumen reduce nitrogen (N) use efficiency (e.g., in g milk N/g of N intake; [1]) and result in high N excretion via urine and feces [2]. Several feeding strategies exist that either aim at reducing rumen degradation of dietary CP [3,4,5] or at more efficient use of the rumen-degradable CP for microbial protein synthesis (MPS) [6,7] in order to increase N use efficiency and to reduce environmental pollution from dairy cattle farming.

For incorporation of available N in the rumen into microbial protein, energy must be available for microbial growth. The supplementation of non-structural carbohydrates as an energy source increases rumen microbial growth and activity and thereby enhances the N incorporation into microbial protein, which, in turn, improves amino acid supply to their host and may enhance overall N use efficiency in lactating dairy cows [7,8]. However, the effects on rumen fermentation and MPS, as well as on animal performance, depend on the amount of supplement, its nutritional composition, and the extent and rate of ruminal starch degradation [9,10]. For instance, the extent and rate of ruminal degradation of starch from oat or wheat differ from that of corn or rice depending on starch granule size, its degree of crystallization, and amylose to amylopectin ratio, among other factors [11,12]. Moreover, synchronizing the amount and time of energy and N supply to rumen microorganisms is considered to maximize the efficiency of microbial protein synthesis (EMPS), and thus the use of CP degradation products in the rumen [13,14], while reducing their ruminal absorption and excretion via urine. Hence, the effects of feeding different types of cereal grains may differ depending on the time when they are offered. Yet, few studies have investigated the effects of timing of supplementation in dairy cows under grazing conditions [15,16].

The objective of the present study was, therefore, to investigate the effects of two cereal grains (i.e., corn and oats) differing in nutritional composition and starch degradation characteristics when they are offered at different times of the day (i.e., before or after grazing) on feed intake, rumen MPS, nutrient digestibility, N partitioning, and milk performance of lactating cows grazing an alfalfa-ryegrass sward. It was hypothesized that the effects of supplementing cereal-based CM will differ depending on the timing of supplementation, but that these differences vary with the cereal species, due to their nutritional characteristics and thus the synchrony in the energy and N supply to rumen microbes. Cereal-based CM were expected to enhance nutrient digestion, N partitioning, and milk performance more effectively when supplemented before grazing than after grazing, in particular, when they contain rapidly degradable starch (e.g., oat grain) instead of slowly degradable starch (e.g., corn grain).

## 2. Materials and Methods

The experiment was conducted between November 2017 and February 2018 at the research station of the National Agrarian University La Molina in Jauja, Peru (11°51′36.3″ S, 75°23′48.8″ W; 3350 m above sea level).

### 2.1. Experimental Design and Animals

Four dietary treatments were tested following an incomplete 4 × 3 Latin square design using four animal groups in three 21-day experimental periods with 14 days of adaptation and 7 days of sampling (i.e., sampling week). Twenty-four lactating Brown Swiss cows (21 multiparous and 3 primiparous) were used. Cows were allotted to four groups of six cows each with similar (arithmetic mean ± one standard deviation) body weight (BW; 458 ± 48.4 kg), days in milk (141 ± 51.9), and milk yield (15.3 ± 1.8 kg/d) at the start of the trial. Each group was randomly assigned to one of the four dietary treatments with each receiving a different diet per experimental period.

### 2.2. Experimental Diets and Feeding

Dietary treatments comprised the feeding of two carbohydrate sources in concentrate mixtures (CM) that were offered in different proportions during morning and evening milking (i.e., the majority of the concentrate was offered either before or after grazing). The CM were prepared every third day by mixing (as-fed basis) 90 kg of either ground corn or ground oats with 15 kg of ground corn cobs, 1.5 kg of calcium carbonate, 1.5 kg of sodium bicarbonate, and 2.1 kg of sodium chloride. Corn and oat were chosen due to their pronounced differences in the extent and rate of ruminal starch degradation with 62% and 6.4%/h for corn and 98% and 15.1%/h for oat, respectively, considering a rumen passage rate of 6%/h [11]. Each cow was fed 3.5 kg/d (as-fed basis) of the respective CM in two unequal portions immediately after the animals entered the milking parlor during the morning and evening milking to create the following four dietary treatments:

- Corn before grazing: 2.5 kg/cow and d in the morning and 1.0 kg/cow and d in the afternoon of the corn-based CM;
- Corn after grazing: 1.0 kg/cow and d in the morning and 2.5 kg/cow and d in the afternoon of the corn-based CM;
- Oat before grazing: 2.5 kg/cow and d in the morning and 1.0 kg/cow and d in the afternoon of the oat-based CM; and
- Oat after grazing: 1.0 kg/cow and d in the morning and 2.5 kg/cow and d in the afternoon of the oat-based CM.

Along with the CM, cows received 100 mL/d of soya oil (Friol^®^ Soya, Alicorp, Lima, Peru) in one dose during morning milking and 50 g of a vitamin-mineral mixture (Engorvisal Leche^®^, Agrofarma International, Lima, Peru; Table 1) at each milking. For this, both, the vitamin-mineral mixture and the soya oil were mixed with the CM just before feeding.

Starting from Day 10 of each period, 5 g of the external fecal marker titanium dioxide (TiO_2_; KRONOS 2300, Kronos International, Westlake, LA, USA) was mixed with approximately 30 g of CM and offered to each cow during both morning and afternoon milking (i.e.,10 g/d TiO_2_ dosages). Once the cow had finished the CM mixed with TiO_2_, the rest of the CM plus the vitamin-mineral mixture and the soya oil were offered. In case of any refusals of the CM, these were recovered in plastic bags before the next cow entered the milking parlor.

Experimental cows grazed together with a herd of 52 lactating dairy cows on an irrigated pasture from 9:30 A.M. to 4:30 P.M. The pasture was dominated by alfalfa (*Medicago sativa* L. cv. Aragon) mixed with ryegrass (*Lolium perenne* L.) and small proportions of white clover (*Trifolium repens* L.) and red clover (*Trifolium pratense* L.). Grazing followed a rationed grazing system. A paddock of approximately 0.60 ha was assigned to the entire herd every day (stocking density during grazing was 114 animal units per hectare with 1 animal unit = 600 kg BW) which was divided into four plots using a solar-powered electric fence. Once the animals finished grazing one plot (vegetation height of ~5 cm), they were moved to the next plot until the cows had grazed the total daily paddock. A total of eight paddocks were used and animals returned to the same paddock every four weeks. Cows did not have access to drinking water on pasture. After grazing, cows were kept in a barnyard from 6:00 P.M. until 6:00 A.M. with access to fresh drinking water, but not to any feed.

### 2.3. Sampling of Feeds and Pasture Forage

One sample each of approximately 100 g (as-fed basis) was taken from the ground corn, ground oat, and corn cobs on Days 15, 18, and 21 during the preparation of the CM of each experimental period. Samples (200 g, as-fed basis) of the CM offered were collected daily during the sampling weeks. At the end of each sampling week, samples of the offered CM and their pure ingredients were pooled per feed type and period by taking the same amount from each individual sample, whereas the total CM refused by each cow in one period was combined to one pool sample. After pooling, two aliquots of 300 g (as-fed basis) per feed type (i.e., ingredients and CM offered and refused) were taken, dried at 50 °C for 48 h, and kept in airtight polyethylene bags until analysis of chemical composition.

Before the cows started grazing, samples of the pasture vegetation were taken every day (i.e., four samples per day) during the sampling weeks by cutting the above-ground plant biomass with hand shears at 5 cm above ground level in a 0.5-m^2^-rectangle (0.5 × 1.0 m). The cutting height of 5 cm was chosen to mimic the vegetation height which animals removed from the plots. The pasture, samples were collected in polyethylene bags and frozen at −20 °C. At the end of each sampling week, the pasture samples were thawed and pooled per day by taking 200 g (as-fed basis) of every sample, resulting in a total of seven pasture samples per period (i.e., one sample per day of sampling week). The pooled pasture samples were then dried at 50 °C for 72 h, ground to pass a 1-mm-sieve (Thomas-Wiley mill 4, 3375-E25, Thomas Scientific, Swedesboro, NJ, USA), and then pooled again by taking 20 g from each sample to make one pooled sample (140 g) per period. The pooled dried samples were subdivided into two aliquots of 70 g each that were kept in airtight polyethylene bags until chemical analysis.

### 2.4. Feeding Behavior

On Day 14 of each experimental period, three cows per group (4 groups × 3 cows = 12 cows) were fitted with noseband sensors (Rumi Watch^®^, Itin+Hoch GmbH, Liestal, Switzerland) before they went out on pasture. The cows wore the halters with the noseband sensors for 8 days every period. The first 24 h of measurements were defined as adaptation time and excluded from the dataset. The data was converted using the RumiWatch Converter V0.7.3.2 (Itin+Hoch GmbH, Liestal, Switzerland) to 24-h summaries of the eating time (min/d), eating chews (n/d), ruminating time (min/d), and ruminating chews (n/d) of each cow.

### 2.5. Spot Sampling of Feces and Urine

Fecal and urine samples were collected every day from Days 15 to 21 in the morning and the afternoon during milking time. Approximately 200 g of fresh matter feces were taken directly from each animal’s rectum and frozen in polyethylene bags at −20 °C. At the end of each experimental period, the fecal samples were thawed overnight, homogenized, and pooled per cow and period by taking 200 g of fresh matter from each daily sample. Pooled samples were homogenized and between 800–1200 g of fresh matter from the pooled samples were taken, dried in a forced-air oven at 55 °C for 72 h, ground through a 1-mm sieve (Thomas-Wiley mill 4, 3375-E25, Thomas Scientific, Swedesboro, NJ, USA), and stored at room temperature until analysis.

Urine samples were collected in plastic buckets by manual stimulation of the perineal area. From the collected urine, 400 mL were passed through a plastic strainer to remove impurities (i.e., hair, feces, or feed particles), acidified with sulfuric acid (20% *v*/*v*) to a pH below 3, and stored at 4 °C in a fridge. Urine samples (200 mL each) from morning and afternoon sampling of the same day were combined. At the end of the sampling period, all urine samples per cow were pooled in a 10-L bucket and mixed thoroughly. Of this pooled sample, two aliquots of 40 mL each were taken and frozen at −20 °C for N analysis. The third aliquot of 40 mL was taken and filtered with filter paper (185 mm pore diameter; Hahnemuehle, Dassel, Germany). From the filtrated urine, 20 mL was diluted in a 100-mL-volumetric flask with distilled water (ratio 1:5; *v*/*v*) and homogenized. Two 10-mL-aliquots of the diluted urine were then taken and frozen at −20 °C until analysis of creatinine and purine derivatives (PD).

### 2.6. Milk Yield and Composition, Body Weight, and Body Condition

Cows were milked twice a day with a SAC milking system (S.A. Christensen and CO, Kolding, Denmark) which consisted of four milking clusters. The milk yield of each individual cow was recorded every day during the sampling week using the analog Waikato MKV milk meter (Waikato Milking Systems LP, Hamilton, New Zealand) with an accuracy of 0.2 kg. One milk sample of 100 mL was collected after milking from the milk meter of every cow once daily from Days 15 to 21, alternating between afternoon and morning milking. The samples were stored at −20 °C until analyses. At the end of each period, the seven milk samples per cow and period were thawed in a water bath at 37 °C. After homogenizing the samples, 50 mL from each day were taken, pooled into one sample per animal and period and 50-mL-aliquots were refrigerated at 4 °C and analyzed for chemical composition. The pooling of milk samples was done irrespective of the daily milk yield because milk yield was similar across the sampling week.

The body weight (BW) was measured with a digital portable scale (315-X6, Pesatec S.A.C., Callao, Peru; accuracy 0.1 kg) before morning milking on two consecutive days at the beginning and the end of each sampling week. The BW of each animal was calculated as the mean of the four BW recorded per period. Immediately after weighing the cows, the body condition score (BCS) of individual cows was evaluated following the scoring table described by Edmonson et al. [17] on a scale of 1 to 5.

### 2.7. Laboratory Analyses

Dry matter (DM) and crude ash concentrations were determined in duplicate for samples of offered CM, pure CM ingredients, CM refusals, and fecal samples by first drying the samples at 105 °C overnight in a forced air-drying oven and then incinerating the dried sample material at 550 °C for 4 h (methods 3.1 and 8.1, respectively) [18]. Afterward, the organic matter (OM) concentrations were calculated [18]. Feed samples and refusals were analyzed in duplicate for neutral detergent fiber (NDF) and acid detergent fiber (ADF) using an Ankom200 Fiber Analyzer (Ankom Technology, Macedon, NY, USA). Alpha-amylase was used for NDF analysis. After NDF analysis, the samples were soaked for 5 min in acetone to remove residual fat (methods 6.5.1 and 6.5.2) [18]. Values of both, NDF and ADF were expressed inclusive of residual ash. Crude lipid concentration was determined in duplicate using the Soxtec system HT 1043 extraction Unit (Gemini b.v., Apeldoorn, The Netherlands) according to method 5.1.1 of VDLUFA [18]. Total N in pasture herbage offered CM, pure CM ingredients, CM refusals, and fecal samples were analyzed in duplicate with the Kjeldahl method (method 4.1.1) [18]. The CP concentrations were then calculated by multiplying the amount of total N by 6.25.

Starch concentrations of the CM and their respective pure ingredients were determined in duplicate according to the polarimetric method 7.2.5 [18] using an enzymatic kit (Test-Combination No. 10 207 748 035, R-Biopharm AG, Darmstadt, Germany).

The gas production during 24-h in vitro incubation was determined for samples of the pasture herbage, the offered CM, and the pure CM ingredients in triplicate in two different incubation runs according to Menke et al. [19]. In vitro gas production and crude nutrient concentrations were then used to estimate the apparent total tract digestibility of organic matter (ivdOM) and metabolizable energy (ME) concentrations of the different feedstuffs using the following equations given by Menke and Steingass [20]:

ivdOM (g/100 g OM) = 9.00 + 0.9991 × GP + 0.0595 × CP + 0.0181 × CA (for corn, oat, and corn cobs)(1)

ivdOM (g/100 g OM) = 15.38 + 0.8453 × GP + 0.0595 × CP + 0.0675 × CA (for pasture herbage)(2)

ME (MJ/kg DM) = 1.06 + 0.157 × GP + 0.0084 × CP + 0.022 × CL− 0.0081 × CA (for corn, oat, and corn cobs)(3)

ME (MJ/kg DM) = 2.2 + 0.136 × GP + 0.0057 × CP + 0.00029 × CL2 (for pasture herbage)(4)

where, GP = net gas production during 24-h in vitro incubation in mL/200 mg DM; CP = crude protein concentration in feed in g/kg DM; CA = crude ash concentration in feed in g/kg DM; and CL = crude lipid concentration in feed in g/kg DM.

All analyses of pasture herbage and feed samples were repeated if the values for duplicate or triplicate determinations differed from the arithmetic mean by >5%. For further calculations, the arithmetic mean of the two aliquots per period of the CM samples was used.

Fecal TiO_2_ concentration was determined in duplicate using the procedure described by Boguhn et al. [21] with slight modifications. The N concentrations in both undiluted urine aliquots per cow and period were determined with the method 984.13 described in AOAC [22]. Urinary allantoin and uric acid concentrations (mmol/L) were determined by spectrophotometry (Agilent Cary 5000 UV-Vis-NIR, Agilent, Santa Clara, CA, USA) in duplicate in both 10-mL diluted urine aliquots following the methods described by Chen and Gomes [23]. Before analysis, the diluted urine samples were further diluted with distilled water to 1:25 (*v*/*v*) for allantoin and 1:20 (*v*/*v*) for uric acid. Xanthine and hypoxanthine are almost absent in cattle urine [24], and therefore were not analyzed. At the same time as the PD analysis, urinary creatinine concentrations were determined in both urine aliquots in duplicate by Jaffé reaction [25]. For this, 1.2 mL of 1% (*v*/*v*) picric acid and 0.20 mL of sodium hydroxide were added in duplicate to 0.4 mL of urine. After 10 min, samples were read by a spectrophotometer (Agilent Cary 5000 UV-Vis-NIR, Agilent, Santa Clara, CA, USA) at 520 nm. Milk samples were analyzed in duplicate for fat, protein, lactose, and urea-N using a Fourier-transform-infrared analyzer (Milkoscan FT 6000, Foss, Hillerod, Denmark).

All analyses of feces, urine, and milk samples were repeated if the results of duplicate analysis differed from their arithmetic mean by >5%. For further calculations, the arithmetic mean of the two urine aliquots per cow and period was used.

### 2.8. Calculations

The apparent total tract digestibility of the organic matter (dOM) of the diet ingested by the cows was estimated from the CP concentrations in their fecal OM using the non-linear regression equation of Lukas et al. [26]. Total fecal OM excretion was estimated from the daily dosage of TiO_2_ and the TiO_2_ concentration in fecal OM assuming a recovery rate of 100% of the external marker in feces [27]. Mean OM intake was estimated from each individual animal’s fecal OM excretion and dOM of the diet consumed by the cows across the sampling week.

The daily OM intake from CM was calculated as the difference between the total amount of CM offered and refused by individual cows during the sampling week divided by seven days. Pasture OM intake was then calculated as the difference between the OM intake of each individual cow and its OM intake from the CM.

The mean daily milk yield of individual cows was calculated across the seven days of the sampling week. Milk protein and fat yields (g/d) were calculated by multiplying their respective concentrations in milk (g/kg milk) by the daily milk yield (kg/d) for each individual cow. Milk N secretion was estimated by dividing the milk protein yield by 6.38. The energy-corrected milk (ECM) yield was estimated using the equation of Orth [28]:

ECM yield = 0.327 × milk yield + 12.95 × fat yield + 7.2 × protein yield(5)

where all yields are in kg/cow and d.

The N balance was calculated by subtracting the total N excretions of individual animals in feces, urine, and milk from their total daily N intake. For this, total N intake (g/d) was calculated as the sum of N intake from pasture herbage and CM. Fecal and urinary N excretions were estimated by multiplying the estimated fecal and urinary output by their respective N concentrations. For urine, urine volumes were estimated from the animals’ mean BW multiplied by an assumed daily urinary creatinine excretion of 29 mg/kg of BW [29] and divided by the creatinine concentration (mg/L) in urine spot samples. The N use efficiency for milk production (g milk N/g N intake) was calculated by dividing the daily milk N secretion by the daily N intake of the respective cow.

Urinary allantoin and uric acid excretions (mmol/d) were calculated by multiplying their molar concentrations in urine by the respective daily urine volume of each animal. Total urinary PD excretion (mmol/d) was calculated as the sum of allantoin and uric acid excretions. Duodenal microbial N flow (g N/d) was then estimated from total urinary PD excretion according to the following equations [23]:

Microbial N (g N/d) = X (mmol/d) × 70/(0.116 × 0.83 × 1000) = 0.727 X(6)

where, 70 = the nitrogen (N) content of purines (mg N/mmol); 0.116 = the ratio of purine N to total N in rumen microbes; 0.83 = the intestinal digestibility of microbial purines; and X = the total amount of purine derivatives (PD) absorbed in the small intestine (mmol/d). X is calculated using the following equation [23]:

X = (Y − 0.385 × BW^0.75^)/0.85 (7)

where, Y = total urinary PD excretion (mmol/d); BW^0.75^ = metabolic body weight of each animal (kg) as kg^0.75^ BW; and 0.85 = the recovery of absorbed purines as PD in urine.

The efficiency of rumen microbial protein synthesis (EMPS) was calculated by dividing the duodenal microbial N flow (g/cow and d) by the digested OM (kg/cow and d). Additionally, ratios between the concentrations of total PD and creatinine or total N in urine were calculated.

### 2.9. Statistical Analysis

The dataset comprised a total number of 72 observations (i.e., 3 experimental periods × 24 cows) and each experimental treatment had a total of *n* = 18 (i.e., 3 experimental periods × 6 cows per treatment) observations. Three values of milk fat concentration, milk fat yield, and ECM, four values of forage intake, dOM, and fecal N excretion, and two values of urinary N excretion were eliminated from the database because milk fat concentrations were either too high (>65 g/kg milk), too low (<20 g/kg milk), or because samples were destroyed or lost (*n* = 4 feces and 2 urine samples) during transport. For animal behavior data, the number of observations was *n* = 9 (i.e., 3 experimental periods × 3 cows per treatment). Additionally, two noseband sensors did not record data in the third period. Data from those cows were treated as missing values in the statistical analysis.

Data were subjected to an ANOVA as an incomplete 4 × 3 Latin square using the Mixed procedure of SAS 9.1 version (SAS Institute, Cary, NC, USA) with the following model:

Yijkl = µ + Ai + Pj + CMk + Tl + CMk × Tl + CMk × Pj + Tl × Pj + CMk × Tl × Pj + eijkl(8)

where, Yijkl = response variable; μ = overall mean; Ai = random effects of each animal i within the group; Pj = fixed effect of period j; CMk = fixed effect of the experimental CM k; Tl = fixed effect of timing of supplementation l; CMk × Tl = fixed effect of interaction between CM k and timing of supplementation l; CMk × Pj = fixed effect of interaction between CM k and period j; Tl × Pj = fixed effect of interaction between timing of supplementation l and period j; CMk ×Tl × Pj = fixed effect of interaction between CM k, timing of supplementation l, and period j; and eijkl = experimental error.

Before analysis, unstructured, autoregressive, compound symmetric, and toeplitz covariance assumption structures were compared. The autoregressive covariance structure was chosen because it showed the smallest Akaike’s information criterion with correction.

Linear contrast comparisons of least-squares means were conducted using the ESTIMATE statement. In the case of non-significant interactions, the main effects of the type of CM and the timing of supplementation were described in the text. Means of both types of CM were compared separately for each timing of supplementation and separately for both timings of supplementation for each type of CM, in case of significant interactions, and superscripts were included in the tables in order to depict any differences. Least-squares means were considered to differ if *p* < 0.05, and tendencies were declared if *p* ≥ 0.05 to <0.10. The degrees of freedom were estimated by the Kenward–Roger method.

## 3. Results

The chemical composition of the ingredients used to prepare the CM (Table 2) showed clear differences in the starch concentration, NDF concentration, and ivdOM. The ivdOM of ground oat and corn cobs was similar, likely because the ground oat grains were not de-hulled and the corn cobs comprised high proportions of grains.

These differences in the chemical composition of the ingredients affected the chemical composition of the CM. While CP concentrations of the oat- and corn-based CM were similar, NDF and ADF concentrations in the oat-based CM were more than twice as high as the respective concentrations in the corn-based CM. In consequence, the ivdOM and concentrations of starch and ME were greater in the corn-based than in the oat-based CM. The nutritional composition of the pasture was relatively constant throughout the experiment; small differences in the nutritional composition between periods were mainly due to differences in the maturity of the pasture between different paddocks.

### 3.1. Feed Intake, Diet Digestibility, and Feeding Behavior

The mean daily OM intake of cows ranged from 12.4 to 12.9 kg/cow and d, which corresponded to 2.6 to 2.7% of their BW (Table 3).

An interaction between the type of CM and the timing of supplementation was observed (*p* = 0.008) for CM intake. The CM intake of cows receiving the majority of the corn-based CM before grazing was lower than that of cows offered the same CM after grazing (*p* = 0.001) or of cows fed the oat-based CM before grazing (*p* < 0.001). No differences in CM intake were observed between timings of supplementation when cows received the oat-based CM (*p* = 0.57). Similarly, CM intake was similar in both CM when they were offered after grazing (*p* = 1.00). There was at least one cow with bloating problems for all the animal groups during the four experimental periods; particularly in the group of cows receiving the majority of the corn-based CM before grazing in period 3, resulting in a lower CM intake of those animals.

There was no effect of the type of CM, the timing of supplementation, or their interaction on total OM intake or OM intake of cows on pasture (Table 3). An effect of CM on dOM was observed with a greater (*p* < 0.001) dOM in cows fed a corn-based CM compared with those fed with an oat-based CM (Table 3). The dOM also tended to be greater (*p* = 0.087) when the majority of the CM was offered before as compared with after grazing.

Daily ruminating time (in min/d) and the number of ruminating chews (both, in n/min of rumination and n/cow and d) were not affected by either the type of CM, the timing of supplementation, or their interaction (Table 3). However, there were interactions between the type of CM and the timing of supplementation for daily eating time (*p* = 0.010) and the number of eating chews (*p* = 0.032). When cows received the majority of the CM before grazing, daily eating time was greater (*p* = 0.046) in cows fed with the corn-based CM than those offered the oat-based CM. Instead, it tended to be greater (*p* = 0.070) in cows offered the corn-based CM than those fed the oat-based CM when the majority of CM was supplied after grazing. Accordingly, daily eating time was also greater (*p* = 0.003) in animals receiving the oat-based CM before as compared to after grazing. While irrespective of the timing of supplementation, the daily number of eating chews was similar (*p* = 0.92) in cows fed the corn-based CM; it was greater (*p* = 0.008) in animals receiving the majority of the oat-based CM before than after grazing.

### 3.2. Milk Yield and Composition, Body Weight, and Body Condition

There were interactions between type of CM and timing of supplementation for daily yields of milk (*p* = 0.075), ECM (*p* = 0.025), milk fat (*p* = 0.035), and milk protein (*p* = 0.013; Table 4). All variables were greater (*p* ≤ 0.052) with the corn-based than the oat-based CM when the majority of CM was offered after grazing, whereas no differences (*p* ≥ 0.29) between CM were found when the animals received the majority of CM before grazing. Moreover, milk protein yield tended to be lower (*p* = 0.066) in cows offered the corn-based CM before as compared with after grazing, while it tended to be greater (*p* = 0.088) in animals fed the oat-based CM when the majority of the CM was offered during morning milking.

There were no effects of CM, the timing of supplementation, and their interaction on urea-N concentrations in milk nor on the BW and BCS of cows.

### 3.3. Urinary Purine Derivatives Excretion and Rumen Microbial Protein Synthesis

Urinary concentrations of allantoin and uric acid (mmol/L) were affected by the timing of supplementation, with greater concentrations of both allantoin (*p* = 0.011) and uric acid (*p* = 0.002) when the majority of the CM was supplemented after as compared with before grazing (Table 5). Statistically, there was no effect of the type of CM or timing of supplementation for total urinary PD excretion and duodenal microbial N flow. However, the above-mentioned variables were numerically greater when the majority of the corn-based CM was supplemented after grazing than before grazing as compared with when the oat-based CM was supplemented before grazing than after grazing. A tendency of an interaction between the type of CM and the timing of supplementation was observed for EMPS (*p* = 0.094) and duodenal microbial N flow expressed as a percentage of the daily N intake (*p* = 0.084). When feeding the corn-based CM after grazing, the EMPS and duodenal microbial N flow as a percentage of the daily N intake of cows tended to be greater (*p* ≤ 0.098) than when feeding the majority of the CM before grazing. No differences (*p* ≥ 0.45) between both timings of supplementation were found when the cows were fed the oat-based CM.

### 3.4. Nitrogen Excretion and Nitrogen Use Efficiency

There were no effects of the type of CM, the timing of supplementation, or their interaction on total N intake, N excretion via urine or feces, and N balance (Table 6). However, an interaction between the type of CM and timing of supplementation (*p* = 0.006) was observed for N intake from CM. The N intake from CM was greater (*p* = 0.001) when the majority of the corn-based CM was supplemented after and not before grazing, but similar (*p* = 0.51) between timings of supplementation for the oat-based CM. Moreover, irrespective of the timing of supplementation, N intake from concentrate was greater (*p* < 0.001) for the oat-based than the corn-based CM. While the N use efficiency was similar for both CMs when their majority was offered after grazing, the efficiency of dietary N use tended to be greater (*p* = 0.098) for the oat-based than the corn-based CM when the majority of both CM was fed before grazing. There were no differences in the ratio of fecal N to urinary N excretion between both timings of supplementation, yet it tended to be greater (*p* = 0.089) for cows fed the corn-based CM compared with those fed the oat-based CM.

## 4. Discussion

The present study analyzed the effects of supplementing grazing dairy cows with corn or oat grain meal, two cereal grains differing in starch concentrations and degradation characteristics, offered before or after grazing. We hypothesized that rumen MPS, nutrient digestibility, N use efficiency, and milk performance of grazing cows are enhanced when CM are offered before rather than after grazing, in particular when a CM containing a rapidly degradable starch is offered (i.e., oat). The evaluated CM not only differed in starch concentration and degradation characteristics but also in NDF, ADF, and ME concentrations. Moreover, CM intake differed between treatments and was lowest when most of the corn-based CM was offered before grazing; although absolute differences were rather small (<200 g OM/d; ~6.5%). Hence, treatment effects cannot exclusively be associated with differences in starch sources or the timing of supplementation.

### 4.1. Feed Intake, Diet Digestibility, and Feeding Behavior

Under the conditions of the present experiment with time-restricted access to pasture (i.e., 7 h/d) and a CM supplementation divided into two meals a day, forage and total feed intakes were similar in cows offered the two types of CM and were not affected by the timing of supplementation. The dOM was lower in cows receiving the oat-based CM compared with those fed the corn-based CM, possibly because of the greater concentrations of NDF and ADF in the former. Timing of supplementation also affected dOM, which, in line with our expectations, was greater when the majority of both CM was offered before grazing, possibly due to the fact that the greater supply of starch as an energy substrate for rumen microorganisms just before grazing enhanced microbial activity, ruminal forage fermentation, and thus dOM [30]. Nevertheless, the differences in dOM were small (769–784 g/kg OM) and even smaller in response to the timing of supplementation than in response to the type of CM.

Despite the lack of differences in forage and total feed intake, daily eating time and the number of eating chews were greater when the oat-based CM was offered before as compared with after grazing. These differences in eating behavior cannot be fully explained but could partly be due to the numerically lower forage and total feed intake of cows receiving the majority of the oat-based CM after grazing, resulting in a similar feed intake rate (g OM/min eating) and bite mass (g OM/eating chew) for both treatments (data not shown). Eating time, however, was also longer in cows offered the oat-based CM than those fed the corn-based CM before grazing, despite the numerically greater forage and total feed intake of the latter animals. Physical distension of the rumen, for instance, at high feed intake levels and/or in response to fiber-rich diets, limits voluntary feed intake and can reduce the grazing time of cows [31]. Nevertheless, the daily feed intake of cows was not very high (~2.6–2.7% of their BW). Moreover, the animals received the same amount of CM and grazed one common pasture and the dOM did not greatly differ between treatments. Hence, although NDF concentrations of the oat-based CM (299 g/kg DM) were greater than of the corn-based CM (108 g/kg DM), pronounced differences in rumen fill and its physical limitation of feed intake rate (e.g., (g OM intake/min eating time)) appear unlikely. On the other hand, propionate such as released during the ruminal degradation of starch is known to reduce appetite and alter the feeding behavior of dairy cows [32]. Daily starch intakes of cows were greater for the corn-based than the oat-based CM (absolute difference of 587 g or 713 g/d when the majority of both CM was offered before or after grazing, respectively). Nevertheless, the supplementation of the oat-based CM as a source of highly and rapidly degradable starch [11,12] just before grazing might have increased ruminal propionate production and thus induced satiety signals during the initial time on pasture. As a result, cows might have reduced their feed intake rate but prolonged their daily eating time in order to be able to maintain their daily feed intake during grazing and to meet their nutrient and energy requirements. In the same line, Oba and Allen [33] observed a lower feed intake rate in diets with low as compared with high starch concentrations, and rapidly as compared with slowly degradable corn starch in lactating dairy cows offered a total mixed ration with forage to concentrate ratios of 43:57 (high starch) or 66:34 (low starch) and with silages of alfalfa and corn as forages.

### 4.2. Milk Yield and Composition

In line with expected differences in dOM, it was hypothesized that milk yield and composition would differ depending on the type of CM and the timing of CM supplementation. There was a significant tendency of significant interactions between CM and timing of supplementation for milk performance variables. The ECM, milk fat, and milk protein yields were greater for the corn-based than the oat-based CM when the majority of these CM was offered after grazing, with no differences between CM when offered before grazing. Additionally, ECM and milk protein yields were greater when the majority of the corn-based CM was offered after than before grazing, while they were numerically or significantly (for milk protein yield) greater when the oat-based CM was offered before as compared to after grazing. These differences may be partly explained by the numerically lower feed intake, dOM, and thus, the intake of digested organic matter (data not shown) of cows fed the majority of the oat-based CM after grazing. Moreover, these interaction effects may also be attributed to differences between CM in their concentrations and rates of carbohydrate degradation which can cause quantitative and temporal imbalances in the N and energy supplied to rumen microorganisms [34].

Starch concentration was greater in the corn-based than the oat-based CM; however, effective rumen degradability of corn starch is lower than of starch from oat grain. Assuming the effective starch degradability reported by Herrera-Saldana et al. [11] of 62% for corn and 98% for oat at a rumen passage rate of 6%/h, the concentrations of rumen-degradable starch and its mean daily intakes across both timings of supplementation were even slightly greater for the oat-based (400 g/kg DM and 1324 g/cow and d) than the corn-based CM (388 g/kg DM and 1240 g/cow and d). Considering that the daily CM supply was divided into two meals, absolute differences in rumen-degradable starch intakes per meal were even smaller (~50 g during the meal when the majority of CM was offered). Cows were turned out on pasture directly after morning milking. Hence, the low solubility and slow rate of degradation of corn compared to oat starch [11,12] may have resulted in a lack of fermentable carbohydrates for rumen microbes when the majority of it was supplemented before grazing, which was probably amplified by the lower dietary starch intake (713 g/d) compared with that of cows receiving the corn-based CM after grazing. By contrast, corn starch degradation likely promoted energy availability and thus microbial growth and activity simultaneous to CP and fiber degradation from the forage ingested on pasture when the majority of this CM was supplemented after grazing. On the other hand, the high solubility and degradation rate of barley starch [11,12] allowed for a temporally synchronous supply of energy to rumen microbes in order to make use of the soluble and rapidly degradable CP, of which the concentrations are quite high in alfalfa and other forage legumes and grasses [35]. These differences between timings of supplementation for the oat-based CM would have likely been more pronounced if no corn cobs had been added to the CM, and thus its starch concentrations had been even greater.

In this line, the numerical differences in duodenal microbial N flow, as well as the tendency of interaction between type of CM and timing of supplementation for EMPS (see next section), suggest that microbial growth and activity were enhanced when the corn-based CM was supplemented after and the oat-based CM fed before grazing, possibly due to improved synchrony in energy and N supply to rumen microbes. Moreover, according to McKay et al. [36], such synchronization in nutrient and energy supply also improves ruminal fiber fermentation, resulting in a greater production of acetic acid and butyric acid as precursors of milk fat, which is supported by the (numerically) greater milk fat and ECM yields for the majority of the corn-based CM supplemented after and of the oat-based CM before grazing. Such differences in ruminal fiber fermentation can be compensated for by microbial fermentation of the structural carbohydrates in the post-ruminal tract, explaining why no interaction effects were found for dOM.

### 4.3. Nitrogen Metabolism and Nitrogen Use Efficiency

The utilization of dietary N can be improved by feeding energy-rich, low-protein supplements to dairy cows grazing high-quality, protein-rich pastures [7,37,38]. For instance, Dickhoefer et al. [7] investigated the effects of supplementing three CM differing in their concentrations of CP and starch to Brown Swiss cows grazing an alfalfa-clover-ryegrass sward using cows from the same experimental farm during a similar season than in the present study. Total and urinary N excretion decreased, and the proportion of ingested N secreted via milk increased with increasing starch and decreasing CP concentrations in their cows’ diets. Compared with the present study, the authors reported a lower total N intake (means across diets of 407 vs. 362 g/cow and d, respectively) but a similar total N excretion (means across diets of 359 vs. 361 g/cow and d, respectively). Nevertheless, concentrations of CP were greater and concentrations of starch similar or even lower in the CM offered by Dickhoefer et al. [7] than in the present experiment. Consequently, cows in the present study excreted less N in urine (means across diets of 41% vs. 53% of total N excretion) and more N in milk (25 vs. 21% of the mean total N excretion) than those in the trial of Dickhoefer et al. [7]. Thus, the efficiency of dietary N use for productive purposes (e.g., g milk N/g N intake) increases when supplementing concentrate feeds or their mixtures rich in non-structural carbohydrates to lactating dairy cows grazing a protein-rich pasture [7].

In this line, it was expected that supplementing CM with rapidly degradable starch (i.e., oat-based CM) would reduce N excretion via urine and enhance N use efficiency in lactating Brown Swiss cows grazing a mixed alfalfa-ryegrass sward, particularly when offered before grazing. Although no significant differences were found in total N intake and fecal or urinary excretion (all in g/d), N secretion via milk (g/d) tended to be greater when the majority of the corn-based was offered after and when the majority of the oat-based CM was offered before grazing. There was also a tendency for more milk N secreted for the corn-based than the oat-based CM when the majority of both were fed after grazing. A similar pattern between treatments in mean ECM, milk fat, and milk protein yields was observed, although differences between both timings of supplementation for each CM were partly not significant (see the previous section). The N use efficiency tended to be greater with the oat-based than the corn-based CM when the majority of those were fed before grazing, confirming our hypothesis.

Both the timing of supplementation and the type of carbohydrate source have been reported to affect rumen microbial protein synthesis and thus total urinary PD excretion [9,39,40]. Hence, the supply of rapidly and highly degradable starch from oat-based CM supplemented before grazing in the present study likely promoted rumen microbial growth, so that a greater proportion of the ammonium produced from rumen degradation of dietary CP was incorporated into microbial protein and was thus available for milk protein synthesis. In line with these results and our expectation, there were tendencies of significant interactions between the timing of supplementation and type of CM for EMPS, which tended to be greater when the majority of the corn-based CM was supplemented after as compared with before grazing, and numerically greater when most of the oat-based CM was offered before instead of after grazing.

Urea-N concentrations in milk and urine N excretion (both, in g/d and g/100 g N intake) were similar across diets, likely because differences in EMPS and microbial N flow (g/100 g N intake) were too small. Moreover, not all of the microbial crude protein digested in the small intestine is converted to milk protein; derivatives of the remainder also contribute to milk urea and urinary N excretion in particular, as energy and not protein was the first factor limiting the milk yield of cows. Hence, the effects of supplementing cereal-based CM and the timing of their supplementation on partitioning in N excretion might be more pronounced in diets in which the protein supply to cows matches their requirements as well as when feeding CM with greater non-structural carbohydrate concentrations than in the present study.

## 5. Conclusions

Interactions exist between the type of CM and the timing of their supplementation related to animal performance, protein metabolism, and N use in lactating dairy cows grazing protein-rich temperate pastures. Hence, the effects of feeding CM, in addition to grazing, that differ in concentrations of starch and/or its rumen degradation kinetics depend on the timing of their supplementation and/or vice versa. Although the effects may be small, supplementing corn-based CM after grazing and oat-based CM before grazing improves EMPS, milk performance, and efficiency of N use in grazing dairy cows on alfalfa-ryegrass swards. Further research is needed, however, to better understand the underlying mechanisms of these interaction effects and to quantify their impact at higher supplementation and performance levels.

## Figures and Tables

**Table 1:** Composition of the multi-vitamin supplement Engorvisal Leche© fed to lactating Brown Swiss cows grazing a mixed alfalfa-ryegrass sward and supplemented with a corn- or an oat-based concentrate mixture before or after grazing.

Ingredient	Concentration (per kg Dry Matter)
Calcium carbonate	250.0 g
Tricalcium phosphate	160.0 g
Magnesium phosphate	15.0 g
Sodium chloride	21.0 g
Sulfur	2.0 g
Zinc oxide	2600.0 mg
Iron phosphate	2200.0 mg
Magnesium oxide	1300.0 mg
Copper sulfate	800.0 mg
Calcium iodate	90.0 mg
Cobalt sulfate	40.0 mg
Sodium selenite	30.0 mg
Cyanocobalamin (B12)	12.0 mg
Vitamin A	500,000 IU
Vitamin D3	90,000 IU
Vitamin E	100 IU

©AGROFARMA INTERNACIONAL S.A.C., Lima, Peru.

**Table 2:** Chemical composition (g/kg DM) of ingredients ^1^, CM ^2^, and pasture herbage offered to lactating Brown Swiss cows grazing a mixed alfalfa-ryegrass sward and supplemented with a corn- or an oat-based CM before or after grazing (*n* = 3 samples).

Parameter	Corn	Oat	Corn Cobs	Corn-Based CM	Oat-Based CM	Pasture Herbage		
						Period 1	Period 2	Period 3
DM (g/kg fresh matter)	971	980	968	948	953	225	221	232
Organic matter	978	958	979	945	944	914	918	919
Crude ash	22	42	21	55	56	86	82	81
Crude protein	86	99	57	80	86	192	251	210
NDF	100	341	481	108	299	316	343	359
ADF	18	162	232	34	127	187	225	218
Starch	668	409	342	625	408	N/A	N/A	N/A
Crude lipid	45	41	23	39	37	13	14	20
ivdOM ^3^	740	660	660	800	670	630	660	590
ME ^3^ (MJ/kg DM)	12.0	10.4	10.3	12.3	10.2	9.0	8.9	7.7

CM, concentrate mixture (i.e., corn-based or oat-based); DM, dry matter; NDF, neutral detergent fiber; ADF, acid detergent fiber; ivdOM, apparent total tract organic matter digestibility estimated based on in vitro incubation; ME, metabolizable energy; N/A, not available. ^1^ Ground ingredients used to prepare the CM. ^2^ The CM contained (as-fed basis) 2.87 kg/d ground corn (corn-based CM) or ground oats (oat-based CM), 0.47 kg/d ground corn cobs, 67 g of sodium chloride, 47 g of calcium carbonate, and 47 g of sodium bicarbonate. ^3^ Estimated from crude nutrient concentrations and gas production during 24 h of in vitro incubation (Menke and Steingass, 1988). For details, please see the text.

**Table 3:** Feed intake, diet digestibility, and chewing behavior of lactating Brown Swiss cows grazing a mixed alfalfa-ryegrass sward and supplemented with a corn- or an oat-based concentrate mixture before or after grazing (least-squares means, standard errors of means (SEM); *n* = 18 ^1^).

Variable (Unit)	Corn		Oat		SEM	*p*-Value			
	Before	After	Before	After		Period	CM	Time	CM × Time
Feed intake (kg OM/cow and d)									
Concentrate	2.9 ^bB^	3.1 ^aA^	3.1 ^aA^	3.1 ^aA^	0.04	0.98	0.008	0.059	0.008
Pasture herbage	9.9	9.6	9.6	9.3	0.71	0.013	0.69	065	0.95
Total	12.9	12.7	12.7	12.4	0.70	0.011	0.79	0.72	0.95
dOM (g/kg OM) ^2^	784	780	772	769	0.3	0.12	<0.001	0.087	0.80
Chewing behavior									
Eating time (min/cow and d)	249 ^aB^	253 ^a^	271 ^aA^	230 ^b^	7.9	0.83	0.96	0.035	0.010
Eating chews (1000 chews/cow and d)	13.0 ^a^	13.0 ^a^	14.1 ^a^	11.8 ^b^	0.49	0.89	0.89	0.042	0.032
Ruminating time (min/cow and d)	379	364	376	376	13.5	0.84	0.72	0.57	0.58
Ruminating chews (1000 chews/cow and d)	28.8	27.5	28.7	28.3	1.04	0.96	0.77	0.44	0.68
Ruminating chews (n/min)	58.6	57.9	58.2	57.6	1.05	0.97	0.74	0.56	1.00

OM, organic matter; dOM, apparent total tract organic matter digestibility. ^1^ *n* = 9 for eating time, eating chews, ruminating time, and ruminating chews. ^2^ Estimated from crude protein concentration in fecal OM (Lukas et al. [26]). ^a,b^ Values within rows for each CM with different superscripts differed (*p* < 0.05) between both timings of supplementation. ^A,B^ Values within rows for each timing of supplementation with different superscripts differed (*p* < 0.05; ^A,B^) between both CM.

**Table 4:** Body weight, body condition score, milk yield, and chemical composition of milk of lactating Brown Swiss cows grazing a mixed alfalfa-ryegrass sward and supplemented with a corn- or oat-based concentrate mixture before or after grazing (least-squares means, standard errors of means (SEM); *n* = 18).

Variable (Unit)	Corn		Oat		SEM	*p*-Value			
	Before	After	Before	After		Period	CM	Time	CM × Time
Body weight (kg)	464	462	473	470	11.8	0.78	0.46	0.79	0.96
BCS	2.36	2.39	2.42	2.47	0.107	0.014	0.52	0.70	0.90
Milk yield (kg/cow and d)	14.8 ^A^	15.6 ^A^	15.1 ^A^	14.1 ^B^	0.50	0.86	0.26	0.85	0.075
ECM ^1^ (kg/cow and d)	14.6 ^A^	16.0 ^A^	15.3 ^A^	14.1 ^B^	0.54	0.56	0.25	0.86	0.025
Milk fat (g/kg)	32.8	33.1	33.6	30.8	1.51	0.21	0.61	0.41	0.31
Milk fat yield (kg/cow and d)	0.45	0.51	0.50	0.43	0.029	0.69	0.53	0.83	0.035
Milk protein (g/kg)	37.4	38.4	39.1	39.0	0.50	0.002	0.020	0.43	0.31
Milk protein yield (kg/cow and d)	0.55 ^A^	0.60 ^A^	0.59 ^A^	0.55 ^B^	0.017	0.45	0.77	0.92	0.013
Lactose (g/kg)	45.0	45.2	45.0	44.4	0.44	<0.001	0.33	0.66	0.38
MUN (mg/dL)	16.7	17.3	16.9	17.2	0.50	<0.001	0.92	0.30	0.78

BCS, body condition score; ECM, energy-corrected milk; MUN, milk urea-nitrogen. ^1^ Calculated using the equation of Orth [28]: ECM = 0.327 × milk + 12.95 × milk fat + 7.2 × milk protein (all in kg/d). ^A,B^ Values within rows for each timing of supplementation with different superscripts differed (*p* < 0.05; ^A,B^) between both CM.

**Table 5:** Urinary purine derivatives (PD) excretion and duodenal microbial nitrogen (N) flow of lactating Brown Swiss cows grazing a mixed alfalfa-ryegrass sward and supplemented with a corn- or an oat-based concentrate mixture before or after grazing (least-squares means, standard errors of means (SEM); *n* = 18).

Variable (Unit)	Corn		Oat		SEM	*p*-Value			
	Before	After	Before	After		Period	CM	Time	CM × Time
Allantoin (mmol/L urine)	8.6	10.3	9.4	10.7	0.57	<0.001	0.27	0.011	0.73
Uric acid (mmol/L urine)	2.5	2.8	2.7	2.8	0.06	<0.001	0.16	0.002	0.57
Total PD ^1^ (mmol/cow and d)	185	207	201	193	11.2	<0.001	0.91	0.53	0.18
PD:creatinine ratio in urine	1.32	1.29	1.29	1.28	0.016	0.001	0.18	0.13	0.61
PD:N ratio in urine	1.28	1.41	1.46	1.37	0.063	<0.001	0.27	0.77	0.100
Absorbed PD ^2^ (mmol/cow and d)	172	198	191	181	12.8	<0.001	0.94	0.51	0.17
Microbial N flow ^3^ (g/cow and d)	125	144	139	132	9.3	<0.001	0.94	0.51	0.17
EMPS (g microbial N/kg digested OM)	13.0	15.9	15.7	14.4	1.23	<0.001	0.62	0.53	0.094
Microbial N (g/100 g N intake)	33.5	41.7	40.4	36.7	3.39	<0.001	0.78	0.51	0.084

EMPS, efficiency of microbial protein synthesis; OM, organic matter. ^1^ Total PD excreted in urine calculated as the sum of allantoin plus uric acid concentrations (mmol/L) multiplied by urine excretion (L/d). ^2^ PD absorbed in the small intestine as estimated according to Chen and Gomes [23]. ^3^ Duodenal microbial N flow as estimated from urinary PD excretion according to Chen and Gomes [23].

**Table 6:** Nitrogen (N) intake, excretion, and N use efficiency of lactating Brown Swiss cows grazing a mixed alfalfa-ryegrass sward and supplemented with a corn- or an oat-based concentrate mixture before or after grazing (least-squares means, standard errors of means (SEM); *n* = 18).

Variable (Unit)	Corn		Oat		SEM	*p*-Value			
	Before	After	Before	After		Period	CM	Time	CM × Time
N intake (g/cow and d)									
Concentrate	40 ^bB^	42 ^aB^	46 ^aA^	46 ^aA^	0.4	<0.001	<0.001	0.058	0.006
Pasture herbage	372	366	368	354	26.8	<0.001	0.76	0.70	0.87
Total	412	408	412	399	26.7	<0.001	0.87	0.75	0.86
N excretion (g/cow and d)									
Feces	131	124	119	113	7.3	0.006	0.13	0.38	0.96
Urine	148	147	149	144	9.0	0.049	0.96	0.74	0.86
Milk	86	93	92	86	2.7	0.44	0.78	0.92	0.014
Total	364	364	364	343	12.9	0.016	0.41	0.39	0.42
Fecal N:urinary N	0.95	0.91	0.80	0.80	0.074	0.11	0.089	0.82	0.81
N balance (g/cow and d)	47	44	43	57	23.2	<0.001	0.87	0.81	0.71
N use efficiency (g milk N/100 g N intake)	22.2	26.0	26.7	24.0	1.80	<0.001	0.48	0.75	0.081

^a,b^ Values within rows for each CM with different superscripts differed (*p* < 0.05; ^a,b^) between both timings of supplementation. ^A,B^ Values within rows for each timing of supplementation with different superscripts differed (*p* < 0.05; ^A,B^) between both CM.

## Data Availability

The data presented in the present study are available upon request from the corresponding author.
